# Efficacy and potential of phage therapy against multidrug resistant *Shigella* spp.

**DOI:** 10.7717/peerj.6225

**Published:** 2019-04-05

**Authors:** Swee-Seong Tang, Sudhangshu Kumar Biswas, Wen Siang Tan, Ananda Kumar Saha, Bey-Fen Leo

**Affiliations:** 1 Division of Microbiology, Institute of Biological Sciences, Faculty of Science, University of Malaya, Kuala Lumpur, Malaysia; 2 Department of Genetic Engineering and Biotechnology, Islamic University Kushtia, Kushtia, Bangladesh; 3 Department of Microbiology, Faculty of Biotechnology and Biomolecular Sciences, Universiti Putra Malaysia, Serdang, Selangor, Malaysia; 4 Institute of Bioscience, Universiti Putra Malaysia, Serdang, Selangor, Malaysia; 5 Department of Zoology, Faculty of Life and Earth Sciences, University of Rajshahi, Rajshahi, Bangladesh; 6 Central Unit for Advanced Research Imaging, Faculty of Medicine, University of Malaya, Kuala Lumpur, Malaysia; 7 Nanotechnology and Catalysis Research Centre (NANOCAT), University of Malaya, Kuala Lumpur, Malaysia

**Keywords:** Multidrug-resistant, Phage therapy, Bacillary dysentery, *Shigella*

## Abstract

*Shigella*-infected bacillary dysentery or commonly known as Shigellosis is a leading cause of morbidity and mortality worldwide. The gradual emergence of multidrug resistant *Shigella* spp. has triggered the search for alternatives to conventional antibiotics. Phage therapy could be one such suitable alternative, given its proven long term safety profile as well as the rapid expansion of phage therapy research. To be successful, phage therapy will need an adequate regulatory framework, effective strategies, the proper selection of appropriate phages, early solutions to overcome phage therapy limitations, the implementation of safety protocols, and finally improved public awareness. To achieve all these criteria and successfully apply phage therapy against multidrug resistant shigellosis, a comprehensive study is required. In fact, a variety of phage-based approaches and products including single phages, phage cocktails, mutated phages, genetically engineered phages, and combinations of phages with antibiotics have already been carried out to test the applications of phage therapy against multidrug resistant *Shigella.* This review provides a broad survey of phage treatments from past to present, focusing on the history, applications, limitations and effective solutions related to, as well as the prospects for, the use of phage therapy against multidrug resistant *Shigella* spp. and other multidrug resistant bacterial pathogens.

## Introduction

*Shigella* is one of the key pathogens responsible for the diarrhoeal disease generally known as bacillary dysentery and more specifically as shigellosis. Until recently, about 55 serotypes belonging to four species of *Shigella* (*Shigella dysenteriae, Shigella boydii, Shigella flexneri* and *Shigella sonnei*) have been identified as being responsible for shigellosis and death worldwide (The et al., 2016). *Shigella* is transmitted efficiently in low doses through direct or indirect human faecal contamination due to poor hygienic conditions (Weissman et al., 1975). Different food products like salads, soft cheese, vegetables and meat products are usually associated with this type of outbreak. Other indirect routes of infections including ingestion of *Shigella*-contaminated food and water, contact via fomites (such as drinking devices, eating utensil and other inanimated objects) and certain modes of sexual intercourse (Morgan et al., 2006; Okame et al., 2012). Different housefly-like vectors which can physically transport infected feaces, are also known to play a vital role in spreading the disease. Antibiotics have so far been the most common therapeutic agents against dysentery. However, the gradual emergence of drug resistant *Shigella* has caused growing concern of the long-term efficiency of antibiotics. The drug resistant characteristics of *Shigella* have been reported since 1940s, and have led to the increasing emergence of multidrug resistant strains over the past few decades. The development of new antibiotics to combat these new strains is time consuming, laborious and costly. Moreover, no effective vaccine is available to prevent shigellosis, which is thus a serious global medical and social problem (Arias & Murray, 2009; Deris et al., 2013; Magiorakos et al., 2012; WHO, 2014).

Increasing antibiotic resistance and the lack of new agents in development make imperative the development of alternative or complementary approaches to antibiotics for treating common bacterial pathogens, including *Shigella*. Currently, phage therapy appears to be a viable option. This therapy has already been utilized in some Eastern European countries with good safety records without significant side effects (Kutter, 2009). For example, phage therapy was successfully used to treat Russian soldiers during and after the World War II (Kutter et al., 2010). Slopek et al. (1987) also reported a 91–100% success rate for cases of phage treatment in Poland against diseases of the digestive system and alimentary tract. In addition, a study in the USA used phages to treat typhoid patients by intravenously injecting phages to modulate immune responses (Desranleau, 1949). No serious side effects were reported. A clinical trial project named ‘Phagoburn’ focused on the efficacy of phage therapy for treating burn wounds patients infected by *Pseudomonas aeruginosa* and *Escherichia coli.* It was the first potential multi-centre, randomised, single blind and well organized clinical trial of phage therapy in the world (Pherecydes Pharma, 2015). This clinical trial was soundly based on Good Manufacturing and Good Clinical Practices and the results gained through the clinical trial indicated significant developments regarding the regulatory framework of phage therapy (Pherecydes Pharma, 2015). Recently, a novel phage therapy has successfully treated patients with multidrug-resistant *Acinetobacter baumannii* infections. The treatment was jointly conducted by the University of California San Diego, School of Medicine, the U.S. Navy Medical Research Center and the Texas A&M University (LaFee & Buschman, 2017).

Phage therapy was originally introduced a century ago, almost 10 years earlier than antibiotic therapy. It has however never outperformed the latter due to several reasons, including the often confusing and inconsistent results of phage therapy trials, the lack of reproducibility, problems in ensuring the administration of appropriate doses, and the limited availability of genetic information on phages (Wittebole, De Roock & Opal, 2014). As a result, many researchers, especially from the USA and Western Europe, gradually lost interest in phage therapy research.

Fortunately, some researchers in Eastern Europe continued to study phage therapy, and performed a significant number of phage therapy trials and treatments (Smith & Huggins, 1983). Although the issue on phage resistant bacteria is a concern, this issue should not be a major concern especially when it is compared with the bacterial resistance to antibiotic. The main reason is because the growth of phages are shadowing bacterial growth, thus they mutate at the same rate as bacteria. Furthermore, with huge number of available phages, there will be certainly another phage or phages that are able to invade the resistant, mutated bacteria (Inal, 2003). Over the years, the advancement of knowledge and technology on phage therapy in Eastern Europe has become the beacon of new hope for exploring the application of phage therapy against multidrug resistant bacteria. The Eliava Institute in Tibilissi, Georgia and the Hirsfield Institute in Wroclaw, Poland are among well-known medical research institutes for phage-based therapeutics.

This review surveys the extent of outbreaks of shigellosis and their effects, and then investigates the treatments of *Shigella* spp. using both antibiotic and phage therapy. It contains a chronological description of the emergence of *Shigella* spp. as a multidrug resistant pathogen, as well as outlining the limitations of antibiotics against multidrug resistant bacterial strains. It goes on to discuss the problems and limitations of phage therapy from the past to the present, together with the recent developments of this therapy as an alternative to antibiotic treatment. It highlights some potential solutions and future directions for the use of phage therapy against drug resistant bacterial pathogens, especially *Shigella* spp. Finally, this review explains why scientists and policymakers should revisit phage therapy, in a positive and progressive manner, in order to find effective cures for drug resistant bacteria.

### Survey methodology

In order to provide a clear picture to readers, we performed a comprehensive literature study covering *Shigella* and shigellosis; multidrug-resistant bacterial pathogens and the related emerging challenges with antibiotic treatments and the development of new antibiotics; and finally the use of bacteriophages and phage therapy against *Shigella* and *Shigella-*like microorganisms. “PubMed”, “Scopus” and “Google” search engines were used to search for journal articles using specific key words: *Shigella*, Shigellosis and phage therapy. Our study describes clearly why and how phage therapy can be a viable alternative or complementary treatment to antibiotics, in particular against *Shigella* and *Shigella-*like organisms. To ensure that our review was comprehensive, logically organized and balanced, we reviewed in chronological order a very broad range of relevant articles published from the time of the discovery of *Shigella* in 1896 up to the present (2018).

#### *Shigella*, Shigellosis and outbreaks

Shigellosis caused by *Shigella* is endemic, and is one of the main causes of mortality and morbidity in all age groups in both developing and developed countries. It is particularly prevalent in children between 0 and 5 years in developing countries (Bardhan et al., 2010; Wen et al., 2012). *Shigellae* are Gram-negative, nonmotile, rod-shaped facultative anaerobic and non-spore-forming bacteria. *Shigella* was first discovered by a Japanese scientist, Kiyoshi Shiga, in 1897 (Shiga, 1898; Trofa et al., 1999). The *Shigella* spp. discovered by him was *Shigella dysenteriae.* The *Shigella* genus was soon expanded with the discovery of *Shigella flexneri* in 1899 (Flexner, 1900), followed by *Shigella sonnei* in 1906 and *Shigella boydii* in 1921 (Barceloux, 2008; Shiga, 1936). These four species of *Shigellae* are further subdivided into different serotypes, based on their type-specific antigens (15 for *S. dysenteriae*; 19 for *S. flexneri*; 20 for *S. boydii,* and 1 serotype for *S. sonnei*) (The et al., 2016).

Currently, *S*. *flexneri* is the main cause of bacillary dysentery in countries with low-income economies, particularly in sub-Saharan Africa and Asia, accounting for almost two third of all *Shigella* infections in these areas. On the other hand, *S. sonnei* is the most common pathogen in high-income or transitional countries, especially in North America and Europe, accounting for up to 80% of all *Shigella* infections in this zone (Gu et al., 2012a). Previously, a multicenter study on Shigellosis conducted in six Asian countries (Pakistan, China, Bangladesh, Vietnam, Thailand and Indonesia) reported *S. flexneri* as the most frequent isolated *Shigella* spp. (68%), except in Thailand (Von Seidlein et al., 2006).

In contrast, shigellosis caused by the species *S. dysenteriae* and *S. boydii* has in recent years been reported in less than 5% cases globally. Interestingly, *S. dysenteriae* was the main cause of dysentery more than 100 years ago, but the incidence of this pathogen is now quite rare (Bardhan et al., 2010; Gu et al., 2012a). In the late 19th and early 20th centuries, *S. dysenteriae* caused numerous outbreaks. It then disappeared for unknown reasons, although *S. dysenteriae* type 1 reappeared as an epidemic in 1968 in Central America, Asia and Africa (Gangarosa et al., 1970; Pal, 1984; Rahaman et al., 1975; Ries et al., 1994). Later, the prevalence of *S. dysenteriae* was replaced by *S. flexneri*, which in turn was gradually replaced by *S. sonnei* (Kostrzewski, 1968; Martin, Pollard & Feldman, 1983). Occurrences of *S. boydii* have meanwhile been reported on the Indian subcontinent and Latin America, but have been infrequent in other regions of the world (Fernandez-Prada et al., 2004; Niyogi, 2005; Rolfo et al., 2011).

Outbreaks of *Shigella* are common, and have been reported widely. For instance, a serious outbreak occurred between 2014 and 2015 in California, with the causative agent being *Shigella sonnei* (Kozyreva et al., 2016). At the same time, the frequency of occurrence and severity of shigellosis outbreaks varied greatly between different regions and countries. In Morobe Province on the northern coast of Papua New Guinea, approximately 1,200 cases and five deaths were reported as shigellosis caused by the *S. flexneri* serotype 2 (Benny et al., 2014), while fifty-five cases of shigellosis were reported in Taiwan caused by *S*. *flexneri* 2a, *S*. *sonnei* and *S*. *flexneri* 3b (Ko et al., 2013). In Bangladesh, a total of 10,827 isolates were identified between 2001 and 2011, with the predominant spp. detected being *S. flexneri,* followed by *S. sonnei, S. boydii* and *S. dysenteriae*, respectively (Ud-Din et al., 2013). In Sichuan Province (China), about 96 students in a rural elementary school suffered from shigellosis after drinking untreated well water; the causal organism identified in this case was *S. flexneri* 2b (He et al., 2012). In another outbreak in Parison city (Iran), 701 inmates experienced severe diarrhea caused by *S. flexneri* serotype 3a (Hosseini & Kaffashian, 2010). Two outbreaks were reported in Sweden in 2009, caused by *S. dysenteriae* (Löfdahl et al., 2009) and *S. sonnei,* that affected air travelers departing from Hawaii (Gaynor et al., 2009). In Austria, a foodborne outbreak of shigellosis was caused by *S. sonnei* (Kuo et al., 2009), and *Shigella* spp*.* (Müller et al., 2009) was reported in Denmark.

The above examples show that outbreaks of shigellosis caused by *Shigella* have been occurring frequently all over the world, from developing to developed countries, with the predominant causative spp. being *S. flexneri* and *S. sonnei*.

#### Multidrug resistant *Shigella* spp.

Multidrug resistant bacterial pathogens impose critical challenge for clinical and pharmaceutical research due to their potentially severe impact on human health. The Infectious Disease Society of America (IDSA) is extremely concerned about the worrying growth in microbial pathogens and antibiotic resistance in the USA and elsewhere in the world (Spellberg et al., 2008). This antibiotic resistance is caused by both bacterial and social factors, such as high mutation frequencies coupled with the exchange of genetic information by bacteria; the misuse or overuse of antibiotics by human beings; and increasing population densities and global migratory movements by animals and people (Huijbers et al., 2015; Liu et al., 2016).

The acquisition of antibiotic resistance in bacteria is due to genetic exchanges via horizontal gene transfer involving three mechanisms (i.e., random transformation, transduction and conjugation). Uptake of small fragments of DNA by bacteria occurs during transformation, while transduction encompasses transfer of DNA (via bacteriophages) from one bacterium into another, and conjugation involves transfer of DNA through sexual pili involving cell-to-cell contact. The newly acquired recipients which were susceptible previously can express resistance due to the resistant genes acquired from the resistant donor (Frost et al., 2005; Oliveira et al., 2017). Moreover, the presence of R factors (plasmids) may play a major role in developing new serotypes which can foster antibacterial resistance (Tanaka et al., 1969). From the beginning of the antibiotic era, tetracycline, ampicillin, chloramphenicol, nalidixic acid and trimethoprim-sulfamethoxazole were used to treat Shigellosis. As *Shigella* developed increasing resistance to these agents, more recently ciprofloxacin, ceftriaxone and azithromycin have served as the mainstays of shigellosis treatments. However, the growing resistances of *Shigella* spp*.* against these antibiotics have also been studied and reported (Table 1) (Klontz & Singh, 2015).

**Table 1:** First use of antibiotics for *Shigella* treatment and initial reporting of resistance.

**Name of drug**	**Beginning period**	**Place and initial report of resistance**	**References**
Sulfonamide	1930s	Philippine Islands (1946) Japan (1952–1957) Israel (1953–1955) USA (1961–1964)	Cheever (1946), Haltalin & Nelson (1965), Marberg, Altmann & Eshkol-Bruck (1958), Mitsuhashi (1969), Mitsuhashi (1971) and Mitsuhashi et al. (1960)
Ampicillin	Late 1960s–1970s	New Zealand (1974) Bangladesh (1974) Mexico city (1976)	Olarte, Filloy & Galindo (1976), Rahaman et al. (1974) and Smith, Bremner & Datta (1974)
Rimethoprim– sulfamethoxazole	1970s	Brazil (1980) Canada (1980) Korea (1981) India (1981) Finland (1975–1982) Bangladesh (1979–1983)	Chun, Seol & Suh (1981), Finlayson (1980), Heikkilä et al. (1990), Macaden & Bhat (1985), Taylor, Keystone & Devlin (1980) and Zaman et al. (1983)
Furazolidone	1970s	Dallas, USA (1972) India (1984)	Bose et al. (1984) and Lexomboon et al. (1972)
Nalidixic acid	1980s	Zaire (1982) India (1984) Bangladesh (1986) Burundi (1990)	Bhardwaj & Panhotra (1985), Munshi et al. (1987), Ries et al. (1994) and Rogerie et al. (1986)
Pivmecillinam	1970s	Bangladesh (2000–2012)	Klontz et al. (2014)
Fluoroquinolone	Late 1980s–1990s	India (1984)	Bose et al. (1984)
Azithromycin	1990s–2000s	India (2006–2011) Netherlands (2012)	Bhattacharya et al. (2014) and Hassing et al. (2014)
Ceftriaxone	1990s–2000s	Korea (2000) Vietnam (2000–2002) India (2006–2011) USA (2003–2012)	Bhattacharya et al. (2014), Pai et al. (2001) and Vinh et al. (2009)

There have been numerous reports of single drug resistance, cross-resistance and multidrug resistance in *Shigella* worldwide, and such cases are growing in both frequency and diversity on a daily basis. In a study, approximately 1,376 *Shigella* isolates were collected from the Foodborne Diseases Active Surveillance Network (FoodNet) and were tested in the US National Antimicrobial Resistance Monitoring System (NARMS) between 2000 and 2010 (Shiferaw et al., 2012). Among the tested isolates, 74% proved to be ampicillin resistant, followed by 58% that were streptomycin resistant, 36% trimethoprim-sulfamethoxazole (TMP-SMX) resistant, 32% sulfamethoxazole-sulfisoxazole resistant, 28% tetracycline resistant, 2% nalidixic acid resistant, and 0.5% ciprofloxacin resistant. Moreover, around 5% of these strains showed multiple resistances to ampicillin, streptomycin, chloramphenicol, tetracycline and sulfamethoxazole-sulfisoxazole (Shiferaw et al., 2012).

In 2002, *S. dysenteriae* type 1 isolates were identified in Eastern India that showed resistance to all available antibiotics, including norfloxacin and ciprofloxacin but with the exception of ofloxacin (Sur, Niyogi & Sur, 2003). In the following year, similar types of isolates were detected in Bangladesh that were resistant to all common antibiotics, including ofloxacin (Naheed et al., 2004). In addition, about 200 *S. sonnei* isolates were identified in Bangladesh that demonstrated a wide range of resistance against frequently used antibiotics, such as ampicillin, mecillinam , ciprofloxacin, nalidixic acid and trimethoprim-sulfamethoxazole, at ratios of 9.5, 10.5, 17, 86.5, and 89.5%, respectively (Ud-Din et al., 2013). More recently, a study in Iran reported high frequency of resistance against trimethoprim/sufamethoxazole, ampicillin, cefotaxime and nalidixic acid (80, 85, 63 and 47%, respectively), in 85 *Shigella* spp isolated from 211 positive stool cultures of children with gastroenteritis (Mahmoudi et al., 2017).

In the annual report of the National Salmonella, Shigella & Listeria Reference Laboratory (NSSLRL-2014, https://www.researchgate.net/publication/280804929), 93% of the 45 *Shigella* isolates listed were identified as multi-drug resistant (Delappe, King & Cormican, 2014). The degree and prevalence of resistance to azithromycin, fluoroquinolones and ceftriaxone do vary considerably between different regions of the world (Bhattacharya et al., 2014). In particular, one study demonstrated that *Shigella* exhibited far higher levels of resistance to nalidixic acid and ciprofloxacin in Asia-Africa than those in Europe-America: 33.6% and 5.0% respectively, or 10.5 and 16.7 times higher (Gu et al., 2012a).

In summary, it is extremely difficult to delimit the geographic range of drug resistant strains of *Shigella* or to control the disease through a single antibiotic, because of the dissemination of resistant pathogens through multiple vectors and the continuous emergence of new serotypes.

#### Medical treatments for Shigellosis

A number of treatments of bacillary dysentery are commonly used. The World Health Organization (WHO) recommends the use of oral rehydration therapy, together with zinc supplements, for 10–14 days. The administration of zinc during shigellosis reduces the duration and frequency of expelling loose stools (Nichter, Acuin & Vargas, 2008; UNICEF & World Health Organization, 2006). The WHO also suggests the use of effective antimicrobials against clinically suspected shigellosis (Christopher et al., 2009). In practice, beta-lactams (amoxicillin, ampicillin, ceftriaxone, cefixime, and pivmecillinam), quinolones (nalidixic acid, ciprofloxacin, norfloxacin, and ofloxacin), macrolides (erythromycin and azithromycin) and other antibiotics (sulfonamides, tetracycline, furazolidone, and cotrimoxazole) are commonly used to treat *Shigella* dysentery. This development, together with the unavailability of the Food and Drug Administration (FDA) approved vaccines, have led researchers to seek alternative treatments against drug resistant bacterial pathogens (Katz et al., 2004; Wu et al., 2011). Administration of antimicrobial peptides and antibiotic cocktails are promising replacements, however, these alternatives may eventually suffer a similar fate as the current treatment (Worthington & Melander, 2013). Conversely, bacteriophages have potentials to be used as an alternative to antibiotics, because phages have different modes of action and they could be rapidly ‘trained’ on ancestral bacterial strains via successive passages, as well as their capability to defeat bacterial resistance by evolving *in situ* mutations (Betts et al., 2013).

Hence, phage therapy could be the best option for treating shigellosis, because it has been shown to work against *Shigella* spp. Phage treatment also has the additional advantage of causing less disruption to gut flora than antibiotic treatment (Kutter et al., 2010). Moreover, experimental anti-dysentery trials using phages have been successfully conducted over several decades in Eastern Europe (Kutter, 2009).

#### Early history of phage therapy

**Figure 1:**
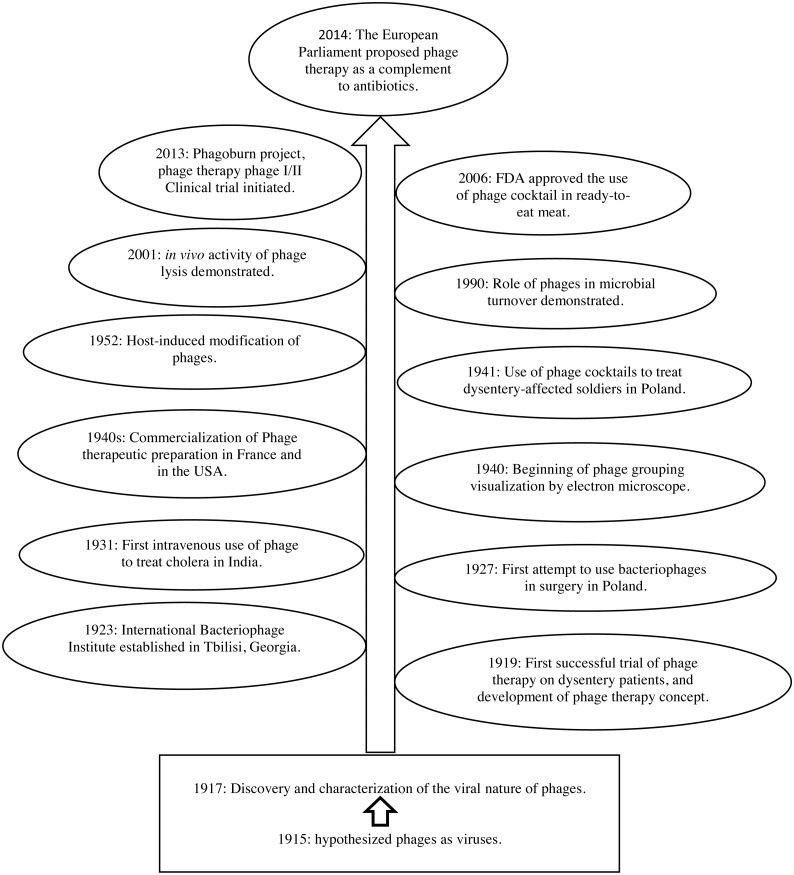
Milestones in phage therapy research, adapted from Elbreki et al. (2014) and Salmond & Fineran (2015).

In the beginning of the twentieth century, Twort (1915) and D’Herelle (1917) independently discovered Bacteriophages (Nobrega et al., 2015). In addition, d’Herelle and his co-workers isolated phages with lytic activity against pathogenic bacteria, including *Shigella* spp*.,* and developed the idea of “phage therapy” meaning the prophylactic and/or therapeutic use of these substances (D‘Herelle, 1923). Bacteriophages were then subsequently used in medicine from 1919 onwards—before the invention of the first antibiotic (penicillin). Figure 1 summarizes important milestones in the development of phage research.

In the early stages, expectations were particularly high with regard to Shiga-phages (phages against *Shigella*), due to their success in treating dysentery patients safely. This success inspired the commercialization of therapeutic phages to treat bacterial infections in humans (Eaton & Bayne-Jones, 1934; Krueger & Scribner, 1941). However, at that time, scientists did not fully understand the mechanisms behind the treatment, and in particular how the phages killed the bacteria. Besides, the outcomes of phage treatment were inconsistent. Moreover, the introduction of antibiotics in the 1940s to treat a broader range of infections led to a reduction in phage therapy research (Matsuzaki et al., 2014). Despite the success of phage therapy in a number of Eastern European countries, it remained largely neglected in Western Europe due to the inconsistent results, the lack of a specific regulatory framework, and the complicated procedures for patenting phages (Verbeken et al., 2014).

#### Current phage therapy

More recently, interest in phage therapy has increased dramatically, and the use of phages in controlling bacterial infections has regained popularity, as well as those unanswered questions of phage therapy are now gradually being addressed (Fischetti, 2001; Stone, 2002; Summers, 2001). By looking back to the pre-antibiotic era, scientists aim to resurrect phages as an antidote to antibiotic resistant pathogens, as well as to solve other medical, agricultural, food safety and environmental problems. Nowadays, the availability of sophisticated molecular tools, the growing understanding of phage control techniques, and the evaluation experiences of Eastern European researchers have all widened the possibility of phage therapy applications. In Eastern Europe, phages have been administered orally (tablets or liquid), topically, rectally and intravenously for almost 90 years with no serious side effects have been reported (Sulakvelidze, Alavidze & Morris, 2001). As a result of these developments, phage therapy has attracted increasing attention as a potential alternative solution in treating antibiotic-resistant bacteria. Six clinics in five different countries (the US, the UK, the Republic of Georgia, Poland and Belgium) are now offering phage therapy for treating diseases (Table 2).

**Table 2:** Main features and activities of Phage therapy centers.

**Name of center**	**Country**	**Main features and activities**
Center for Phage Therapy	Poland	Since 1980, specific bacteriophages have been used to treat over 1,500 patients with suppurative bacterial infections, where routine antibiotic therapy has failed (http://www.iitd.pan.wroc.pl).
Eliava Phage Therapy Center	Georgia	A network of eight laboratories have developed bacteriophage preparations for fighting against dangerous and antibiotic-resistant superbugs (http://www.mrsaphages.com).
Novomed	Georgia	Effective treatment delivery through phage therapy in many areas of medicine, drawing on the expertise of local physicians. Treatments are available not only to local Georgians, but also to foreign patients, especially those with chronic wounds, osteomyelitis or other types of acute and chronic infections (http://www.phagetherapy.com).
Phage Therapy Center	Georgia	Provides excellent treatment for patients who have bacterial infections and are difficult/non-healing, chronic, drug-resistant or have not responded to conventional antibiotic therapies (http://www.phagetherapycenter.com).
Phage International Inc.	The United States	Treats patients with chronic, drug-resistant or difficult to treat infections (http://www.phageinternational.com)

There are 11 US and international biotechnology companies, as well as a number of academic investigators currently working in the field of bacteriophage technology and products. These companies and researchers are utilising bacteriophages in the food processing industry and for the treatment of human diseases. For example, US companies such as Intralytix and Novolytics are using bacteriophages as biotechnology tools and as platform technologies (http://www.dreamingrock.com/viridax/eviridax/cphage.htm). The FDA and the United States Department of Agriculture (USDA) have marked a milestone in phage research by approving three phage products, each comprising a “cocktail” of phages, to target and kill bacteria. The first FDA-approved phage product was ListShield™ (Intralytix, Baltimore, MD, USA) which can be used as a food additive against *Listeria monocytogenes* in ready-to-eat meat and poultry (Mead et al., 2006). The second product, EcoShield™ (Intralytix, Baltimore, MD, USA), can be sprayed on red meat, in order to kill *Escherichia coli* (particularly strain O157:H7) before the meat is ground into hamburgers (Abuladze et al., 2008; Scallan et al., 2011). The third phage product, called SalmoFresh™ (Intralytix, Baltimore, MD, USA), which acts against *Salmonella enterica* was approved as a food processing aid. It is used for the treatment of fresh and processed vegetables, fruits, seafood such as shellfish and fish. Lately, another phage preparation- ShigaShield™ (Intralytix, Baltimore, MD, USA), is currently undergoing FDA and USDA reviews for the GRAS (Generally Recognized As Safe) status (GRN672). According to the report by Soffer et al. (2017) this Shigella phage product, ShigaShield™ is able to reduce Shigella levels in various foods experimentally contaminated with a *S. sonnei* strain. Novolytics has the aim to lead in the utilisation of bacteriophage as a treatment for bacterial infections. Currently, the company’s most promising products NOVO12, a phage cocktail, administered as a form of gel for topical treatment of MRSA (Methicillin-resistant *Staphylococcus aureus*) infections (http://www.cobrabio.com/News/June-2013/Cobra-Biologics-and-Novolytics-Unveil-Successful-D).

The European Union (EU) also shows support for phage therapy research. In 2013, a project entitled ‘Phagoburn’, aimed at exploring the efficacy of phage therapy in protecting patients of burn wounds against severe bacterial infection, was funded by European Commission (Matsuzaki et al., 2014). In April 2014, the European Parliament passed a resolution in favour of prioritizing the development of phage therapy as a complement to antibiotic therapy in order to combat antibiotic resistance (Council of Europe, 2014). This is an important milestone fostering phage therapy research and development, but more time is needed to see its practical impact in future.

#### Phage biology and phage-host interaction

Bacteriophages exhibit four known life cycles inside the bacterial host: lysogenic, pseudo-lysogenic, chronic and lytic infection (Drulis-Kawa et al., 2012; Weinbauer, 2004). For phage therapy, the main interest has always been focused on lytic phages particularly the families of *Podoviridae*, *Myoviridae* and *Siphoviridae*. There are also a few reports on the applications of filamentous phages and cubic phages in phage therapy (Drulis-Kawa et al., 2012). For any type of life cycle, the initial step of an infection is the recognition and binding of a phage to a host receptor, which is facilitated by the phage receptor binding protein (RBP). The host specificity of bacteriophages towards different bacterial cells depends on different RBPs (Le et al., 2013). Three types of host receptors in *Salmonella* were identified by Shin et al. (2012): flagella, BtuB (outer membrane protein up taken by vitamin B12) and lipopolysaccharide-related O-antigen. Transmission electron microscopy analysis showed that the phages from *Podoviridae* family use O-antigen of LPS as a receptor while phages from *Siphoviridae* family use flagella (BtuB) as a receptor. Most frequently, mutations of these receptors caused the host cells resistant to these phages (Shin et al., 2012). The recognition of phage to host cells and the subsequent binding of RBPs and host receptors stimulate a spectrum of the probable phage-bacteria interactions (Wittebole, De Roock & Opal, 2014). After binding to host receptors, phages inject its DNA into the host cell via inducing a pore in the host cell wall and leaving behind their capsids outside the bacterial cell. Before lysis of bacterial cell occurs, packing and assembly of phages take place. Finally, release of phage progenies from the hosts. Different phage enzymes (murein synthesis inhibitors, lysins and holins) are then involved for helping the release of phage progenies into the extracellular environment (Weinbauer, 2004). In molecular aspect, when a phage invades a susceptible bacterial cell, its nucleic acid enters the cell and induces production cycle of the phage. The cell is converted to a phage factory. Some of the components of the biosynthetic apparatus involved in bacterial growth and metabolisms (such as ribosomes and ATP generators) are no longer performing their normal tasks during the phage production cycle (Campbell, 2003). It is known that while bacteria can evolve to become resistant to phages, phages can also develop new mechanisms to infect the resistant bacteria. Hosseinidoust, Tufenkji & Van De Ven (2013) demonstrated that resistance development is linked to changes in bacterial fitness and alteration of virulence determinants that are usually maintained in the absence of the agent to which the bacteria confer resistance. The alteration of phenotypic characteristics is associated with changes in gene regulation levels.

#### Phage therapy *vs* antibiotic treatment

Treating multidrug resistant *Shigella* spp. by a new antibiotic or a new combination of antibiotics tends to be more complicated than treating it with a phage or phage cocktail (Khatun et al., 2011). Generally, phages are environmentally friendly. In both cases, the clinical trial is expensive but it is usually quicker and less expensive to select, isolate and identify phages than to develop a new antibiotic, which can take a longer period (Matsuzaki et al., 2005; Weber-Dąbrowska, Mulczyk & Górski, 2001). Secondary infections may happen but very rare and minimal in phage therapy compared to antibiotics. The term secondary infection during phage therapy is due to the interaction between phage and bacteria, which can cause superinfection immunity or superinfection exclusion. Alternatively, secondary infection can also be described equivalently to superinfection or coinfection, which can result in phage-on-phage parasitism, genetic exchange between phages as well as various partial reductions in phage productivity that have been termed as partial/mutual exclusion, or the depressor effect (Abedon, 2015). With respect to antibiotics, it can cause secondary infection by attacking the normal flora of patients, in addition to the targeted pathogens (Table 3). In addition, phage resistance is less of a concern than antibiotic resistance, because phages can mutate and evolve naturally to counter phage-resistant bacteria (Ho, 2001; Matsuzaki et al., 2005). Moreover, the phage resistance development can be mitigated by using phage cocktails (combinations of multiple phages) and/or by applying phages in conjunction with antibiotics as therapy (Ho, 2001; Kutateladze & Adamia, 2010). The differences between phage therapy and antibiotic treatments are summarized in Table 3.

**Table 3:** Comparison between phage therapy and antibiotic treatment.

**Feature**	**Phage**	**Antibiotic**
Host specificity	Very specific to their host cells: usually affect primarily or exclusively the targeted bacterial species (Chernomordik, 1989).	Can target a wide range of pathogenic microbes. Can therefore be used when the exact disease-causing pathogen is unknown. However, this can lead to the emergence of new drug resistant pathogens (Sulakvelidze, Alavidze & Morris, 2001).
Mode of action	Bacteriophages replicate exponentially as long as the specific bacteria they are targeting are available in abundance. They replicate at the site of infection and are available where they are most needed (Smith & Huggins, 1982).	Antibiotics are metabolized and then expelled from the body, and do not necessarily concentrate at the site of infection (Sulakvelidze, Alavidze & Morris, 2001).
Side effects	Generally the side effects are less than the antibiotic treatment. No or very few side effects have been described (Sulakvelidze, Alavidze & Morris, 2001).	Due to their non-specificity to the host, antibiotics destroy commensal microflora. This can lead to several side effects, including allergies, intestinal disorders and secondary infections (Inal, 2003; Lehmann, 1999).
Time and cost for new development	The selection of new phages against drug resistant or phage resistant bacteria is a comparatively rapid process which can be carried out in days or weeks (Sulakvelidze, Alavidze & Morris, 2001). Sometimes, it also takes longer period and extra cost for safety approval and *in vivo* trial.	The development of a new antibiotic against antibiotic resistant bacteria is not only time-consuming, but can also cost millions of dollars for clinical trials, and so may not be cost-effective (Chopra et al., 1997; Silver & Bostian, 1993).
Dose administration	Repeated dose administration is not always essential , because the phage reproduces until the target bacterium is destroyed (Inal, 2003).	Most cases require repeated dose administration.
Application range	In spite of some negative effects, the range of applications of bacteriophages is broader: they can, for example, be applied as protective materials in food supplements, the milk industry, pharmacology, toothpastes, cleaning solutions and so on (Veiga-Crespo & Villa, 2010).	The application ranges of antibiotics are restricted and narrower.

One of the advantages of phages is that they have much fewer side effects. In fact, the prolonged use of phages to treat human infections in Eastern Europe has not elicited any allergic reactions, nor have animal trials in Western Europe revealed any unusual histological changes, mortality or morbidity when phages were administered orally, intravenously or intramuscularly (Biswas et al., 2002; Carlton et al., 2005; Merril et al., 1996). Indeed, intakes of the T4 phage up to 10^5^ PFU (Plaque Forming Unit) have not caused any secondary effect (Bruttin & Brüssow, 2005). The intravenous injection of purified phages has not produced any side effect in either HIV-infected individuals (Fogelman et al., 2000), healthy volunteers (Ochs et al., 1993), or other patients with immunodeficiency diseases (Ochs, Davis & Wedgwood, 1971). Phage therapy has been successfully used to treat antibiotic resistant infections in the Southwest Regional Wound Care Centre in Texas (Clark & March, 2006), while biodegradable patches impregnated with phages have also been applied to patients with prolonged infections in Georgia (Fischetti, Nelson & Schuch, 2006).

In summary, as antibacterial agents, phages have a number of properties that make them a compelling alternative to antibiotics. Moreover, most of the concerns associated with phage therapy should be manageable through a combination of appropriate phage selection, effective formulations, and clear knowledge and expertise on how to prepare and apply phages (Loc-Carrillo & Abedon, 2011).

#### Phage therapy for controlling *Shigella*

There is a historic relationship between *Shigella* spp. and the discovery of phages. The first application of phage against human infections was conducted by d’Herelle in 1919 to treat the symptoms of dysentery. He injected an anti-dysentery phage into a patient with severe dysentery (10–12 bloody stools per day). The patient made a rapid recovery, displaying no symptoms shortly after receiving the phage therapy (Summers, 1999). This pioneering experiment of d’Herrelle led to many successful applications of this therapy against dysentery, which were reported in scientific articles over the subsequent 20 years. For instance, in the US state of Maryland, *Shigella flexneri* was identified in dysentery-affected children, and phage therapy was given orally and rectally in doses ranging from 5 to 1,300 ml (Davison, 1922). In one successful example, Spence & McKinley (1924) treated shigellosis patients through the oral administration of 10 ml phages, which substantially reduced their mortality rate and length of stay in the hospital (10% and 5.8 days, respectively) when compared to a control group in another hospital (40% and 12.8 days). Another example, Querangal des Essarts (1933) treated a bacillary dysentery patient in France with a polyvalent Shiga-Flexner bacteriophage through the oral administration of 5–10 ml of phages with alkaline water during an outbreak on board two ships at the port of Brest in 1933. The results were remarkable, with blood and mucus rapidly ceased (2nd or 3rd day) and the stools reverted to normal on the 4th day. The same physician also stopped an outbreak of dysentery by the prophylactic administration of bacteriophages among newborns at a holiday camp (Goodridge, 2013).

On the other hand, there have also been some failures, mainly due to the late administration of the therapy. Vaill & Morton (1937) reported that out of 200 cases of dysentery treated with bacteriophages in New Jersey (USA), only 22 cases were successful (Vaill & Morton, 1937). Johnston, Ebbs & Kaake (1933) treated 70 infants aged less than 2 years old using 1 ounce of bacteriophage per hour, and found that the clinical course of dysentery was not improved as what they expected with this therapy. These lower success rates may be due to the fact that the trials used a strain-specific bacteriophage, and the phage only effective against 17 out of 94 bacterial strains which is approximately 20% of bacterial strains tested *in vitro* (Johnston, Ebbs & Kaake, 1933). The British army conducted a phage therapy research in the Middle East and the experiment was divided into four small scales, of which two were reported as unsatisfactory results. The unimpressive result of the third one was published in the British Medical Journal. The last experiment was administrated judiciously and among the 32 enrolled cases, the control cases, and phages treatment cases were 18 and 14, respectively. The outcomes of this research did not show any remarkable result but a marginally better improvement of the treated cluster than the control cluster (Boyd & Portnoy, 1944; Goodridge, 2013).

Nonetheless, phage therapy has been successful in most cases. In 1938, Haler reported the phage treatment of a dysentery epidemic caused by *Shigella sonnei* in which the patients were administered with bacteriophages three times daily and the epidemic ceased after two days and no further case was observed for a year (Haler, 1938). In Poland (1941), 10 ml of local phage mixture containing sodium bicarbonate in a half cup of tea or coffee was found effective against *Shigella* infection (Kliewe & Helmreich, 1941). It has also been shown, in 1945, that the effective proportion of a phage can be diluted up to a 1:10 ratio of phage-bacterium injection. In a bacterial challenge experiment, mortality can be prevented with phage treatment up to 4 days before the challenge or with maximum 3 h delay after the challenge (Morton & Engley Jr, 1945). In 1957, the Hirszfeld Institute of Immunology and Experimental Therapy (HIIET) in Poland applied phages to treat shigellosis and other infectious diseases caused by antibiotic resistant bacteria, which were untreatable by conventional antibiotics (Sulakvelidze, Alavidze & Morris, 2001). In the 1960s, a clinical trial was conducted extensively to evaluate the efficacy of phage therapy against shigellosis (Babalova et al., 1968). This study was performed in Tbilisi, Georgia in which 30,769 children were involved. The children, aged between 6 months to 7 years old were divided into two groups, with one group being given tablet made of dried *Shigella* phages and the other group a placebo, orally once a week, for each child. These children were monitored for 109 days and the results showed that the occurrence of dysentery was nearly a fourfold higher in the children given placebos than those treated with phages (Babalova et al., 1968). In another investigation reported in 1984, Anpilov & Prokudin (1984) demonstrated that the phage-mediated preventive treatment of shigellosis produced a ten-fold reduction in the incidence of dysentery among the phage-treated patients. Miliutina & Vorotyntseva (1993) conducted an experiment on phage therapy and a combined phage-antibiotics treatment on shigellosis and salmonellosis in 1993. They observed that the combined phage-antibiotics treatment was more effective in some cases as compared to the antibiotics treatment alone.

There are many articles reporting successful treatments of Shigellosis in 21st century. The efficacy of phages against multidrug resistant *Streptococi* and *Pseudomonas* as well as some antibiotic resistant *Enterobacteriaceae* family members, including the genera of *Shigella*, *Salmonella*, *Serratia*, *Escherichia*, *Klebsiella* and *Proteus*, have been investigated (Kumari, Harjai & Chhibber, 2010). These studies have largely confirmed the viability of phage therapy as a treatment for gastrointestinal distress, particularly for *Shigella*. Zhang, Wang & Bao (2013) studied the ability of *Shigella-*specific phages and phage cocktails to inhibit *Shigella* spp in chicken products. They concluded that phages with higher concentration (3 × 10^8^ PFU/g) could lyse bacteria more effectively in comparison to phages with lower concentration (1 × 10^8^ PFU/g), and that the *Shigella-*specific phages were able to significantly reduce or eliminate *Shigella* spp. in the edible chicken products.

In summary, from the very beginning to the present day, the success rate of phage therapy against Shigellosis has been promising. An intensive and extensive studies of anti-*Shigella* phages could therefore help to identify alternative treatments for the increasing number of drug resistant bacteria, and hence reduce the pressure to find new antibiotics. In the longer term, greater use of phage therapy could help to reduce the emergence of new multidrug resistant bacterial strains.

#### Limitations, solutions and prospects for phage therapy

Despite all the advantages of phage therapy, it is still a long way from being the “magic bullet” for treating infections, because many parameters (e.g., frequency and duration of treatment, route of administration and optimal dose) have yet to be determined precisely through clinical trials (Wittebole, De Roock & Opal, 2014).

The major limitations of phage therapy are summarized below based on the reports from a few research groups (Hermoso, García & García, 2007; Kutter & Gowrishankar, 2001; Matsuzaki et al., 2014; Nilsson, 2014):

I. A narrow host range as well as serotype specificity (which might reduce effectiveness and coverage).
II. A single phage is inadequate for treating illnesses caused by multiple bacteria.
III. The release of various pro-inflammatory components (endotoxins and peptidoglycans) from bacteria lysed by phages might cause problems in the human body.
IV. There is a possibility that resistant bacteria might emerge after treating with phages, however phages can evolve and adapt to combat resistant bacteria.
V. Complicated pharmacokinetics and pharmacodynamics of phage treatments and interference by anti-phage antibodies.

In addition, there are other problems associated with patenting, manufacturing, and administration which often create obstacles for development of phage therapy. The lack of a definite regulatory outline reflecting individualized therapies, or difficulties for the pharmaceutical companies to register intellectual properties for phage and phage products are some of the major problems in phage therapy (Nobrega et al., 2015; Young & Gill, 2015). The eventual success of phage therapy will largely depend on the development of appropriate strategies to overcome these limitations. In addition, adequate regulatory framework must be created, appropriate safety protocols have to be implemented and the general acceptance of public towards phage treatment is needed (Nobrega et al., 2015).

Several initiatives have been taken to overcome the limitations in phage therapy. Phage cocktails have been formulated, consisting of several phages with complementary features (different receptors) which can play a vital role to overcome the limitations of a single phage with its narrow host range (Chan & Abedon, 2012; Chan, Abedon & Loc-Carrillo, 2013; Goodridge, 2010). In addition, phage cocktails containing different types of phages potentially capable of combating the same species and strains of bacteria could reduce the emergence of bacteria resistant to phage (Chan & Abedon, 2012; Gu et al., 2012b; Potera, 2013). A complementary approach proposed by Friman et al. (2016), where phage cocktails can also be modified by including not only various phages, but also *in vitro* evolved phages from different evolutionary time points (Friman et al., 2016). Moreover, the host range of phages can be broadened by engineering their genomes to express endosialidase (Ackermann, 2001) and by substituting the gene encoding putative host binding proteins (Yoichi et al., 2005). In addition, the challenges of phage therapy may be overcome by producing genetically modified phages (recombined-phage genomes, site-directed mutagenesis, selected spontaneous mutants or phage display techniques) (Chhibber & Kumari, 2012; Dąbrowska et al., 2014; Moradpour & Ghasemian, 2011). Mutated phages could also be used to overcome bacterial resistance as well as to prevent the human immune system against phages (Matsuzaki et al., 2014).

The efficacy of phage therapy could be enhanced by utilizing the antimicrobial synergy between phages and antibiotics. Torres-Barceló et al. (2016) found a strong synergistic effect on bacterial population density by applying treatment with combination of antibiotics and phages. Their results indicated that phages not only could contribute in managing the level of antibiotic resistance but also limit the consequences of bacterial virulence evolution. In another study with experimentally challenged mice, Mai et al. (2015) demonstrated that a combination of phage cocktail (5 *Shigella* specific bacteriophages) and an antibiotic (ampicillin), designated as ShigActive™, was able to decrease *Shigella* counts effectively. They did not observe any deleterious side effects of phage application during this study, and the impact of phage cocktail on the normal gut microbiota was much lesser than that caused by the treatment with generally recommended antibiotics (Mai et al., 2015).

This synergistic effect could hasten cell lysis and allow phages to spread more quickly (Comeau et al., 2007; Ryan et al., 2012). Thus antibiotics conjugated to phages could enable the delivery of antibiotics to specific cells and cause an increase in local drug concentrations (Yacoby & Benhar, 2008). At the same time, the antibiotic resistance of bacteria could be minimized by applying phages to inject sensitizing alleles of the mutated genes (e.g., *rpsL* and *gyrA*) for restoring drug efficacy. For instance, temperate phages have been used to reverse antibiotic resistance of pathogenic bacteria by lysogenizing the genes *gyrA* and *rpsL* in which both conferred sensitivity in a dominant fashion to two antibiotics, nalidixic acid and streptomycin, respectively. This made the bacterial pathogens sensitive to antibiotics prior to host infection (Edgar et al., 2012). In addition, the incorporation of genes that inhibit stress responses, improve drug uptake or repress biofilm production can enhance the antibiotic sensitivity of *E. coli* (Lu & Collins, 2009).

A number of foodborne pathogens from the family *Enterobacteriaceae* including *Shigella*, contain prophages which encodes the Shiga-like toxin, a major virulence factor. In *S. flexneri* the O-antigen modification (serotype conversion) is a key virulence determining factor, which is introduced by temperate bacteriophages (Allison & Verma, 2000). A careful screening of the phage genome for virulence genes would help to minimize the risk of phage engineering. Another approach for the safe use of phage therapy is to use the viral gene products (endolysins) instead of the whole phage particles (Fischetti, 2005; Nelson et al., 2012; Schmelcher, Donovan & Loessner, 2012). The use of gene products might eliminate the risk of phages giving toxic properties to bacteria (Hermoso, García & García, 2007) and thus reduce the risk of resistance developing (Borysowski, Weber-Dąbrowska & Górski, 2006; Nelson et al., 2012; Schmelcher, Donovan & Loessner, 2012).

Another important limitation of bacteriophage therapy is the capability of phages to act only on outside of bacterial cell and the risk of being attacked by the *in vivo* anti-phage antibodies (Singla et al., 2016). To overcome these risks, Singla et al. (2016) used liposome as a delivery vehicle for phages. This study reinforced the growing interest to apply phage treatment as a means to target multiple drug resistant (MDR) bacterial infections, as the encapsulation of phages has increased the efficiency to overcome most of the difficulties and problems related to the clinical use (both *in vitro* and *in vivo*) of phages (Singla et al., 2016).

As things now stand, most of the drawbacks of phage therapy have been overcome to a lesser or greater degree, and phages are now capable of being successfully incorporated into the era of multi drug resistant treatment. As further steps, next-generation sequencing could be employed to determine genomic DNA sequences from multiple phage products, which could reduce further the risks of phage therapy by eliminating harmful genes and gene products (Matsuzaki et al., 2014). Recently the whole genomes of all five lytic bacteriophages of the cocktail ShigaShield™ have been sequenced and analysed, and no undesirable genes have been found, including those listed in the US Code for Federal Regulations (40 CFR Ch1) (Soffer et al., 2017). In addition, the multi-route administration of phages (intramuscular, intravenous, intraperitoneal, subcutaneous, intranasal and oral) would broaden the use of phage therapy as a potential agent in the future. Moreover, the prophylactic use of phages and the development of vaccines using phages or phage products would open up a new dimension for the prevention of antibiotic resistant pathogens (Chanishvili, 2012; Morello et al., 2011). In addition, the active participation of dysentery patients and a large scale trial of phage therapy against multidrug resistant shigellosis and other dysenteries would enhance the acceptance of phage therapy as a common treatment. Finally, it is essential to build up public awareness of phage therapy as well as expand the availability of phages and phage therapy centres in order to expand and exploit this potentially fruitful innovation.

## Conclusion

Phage therapies for *Shigella* spp. and other pathogenic bacteria have been studied and applied for about a century, but phage therapy as an antibacterial treatment in general has not received much attention due to lack of clinical knowledge and public awareness of phages. However, given that the development of novel antibiotics is laborious, time-consuming and costly, it makes eminent sense to seek alternative antimicrobial approaches to combat drug resistant pathogens. While it inevitably has some drawbacks, phage-based biocontrol and bacteriophage therapy are very promising approaches to combat the challenge of pathogenic bacterial infections, particularly when the search for new antibiotics is stagnating. The potential of phage therapy has been acknowledged and revisited by many scientists over the last few decades, and there has been a rejuvenation of research into phage therapy. Moreover, phages have many unexploited potentials as an alternative to antibiotics, both due to the range of intrinsic variation in their mode of action, also due to almost unlimited variety of phages and their ability to evolve *in situ* to successfully deal with bacterial resistance. The FDA has approved bacteriophages as GRAS and allowed the application of phages as food additives in 2006, which is a significant boost to phage therapy research.

Nevertheless, the therapeutic application of phages still requires extensive studies, judiciously performed clinical trials, and importantly well-defined regulatory guidelines. Currently, phage therapy is encouraged in many parts of the world because policymakers consider growing MDR as a serious health problem. This awareness should further encourage researchers to study the biological properties of phages, which eventually increases their safety and efficacy. Furthermore, genetically modified phages could help to solve the issues of patent filing and as a result increase the interest of pharmaceutical and biotechnology companies to produce phage-products. Finally, cocktails of natural phages and genetically modified phages could open new perspectives for successful phage therapy in the future, particularly against the major challenge of *Shigella* and *Shigella-*like multidrug resistant bacteria.

## Additional Information and Declarations

### Data Availability

The following information was supplied regarding data availability: This is a literature review article. No raw data was generated.
